# Ileal Conduit Peristomal Varices Treated via Percutaneous Transhepatic Embolization: A Case Report

**DOI:** 10.7759/cureus.109668

**Published:** 2026-05-26

**Authors:** Lelde Tiltina, Tiago Pereira

**Affiliations:** 1 Radiology, Riga East Clinical University Hospital, Riga, LVA; 2 Radiology, Riga Stradins University, Riga, LVA; 3 Radiology, Trofa Saúde, Vila Nova de Gaia, PRT

**Keywords:** ileal-conduit, interventional radiology-guided embolization, peristomal varices, portal hypertension, stomal hemorrhage

## Abstract

The development of ectopic varices is a well-documented but relatively rare consequence of portal hypertension. While the majority of portosystemic shunting in cirrhotic patients occurs at the gastroesophageal junction, some patients will develop varices in ectopic sites, like peristomal varices, which are a unique subset of ectopic varices. In this specific case, the patient’s history of a Bricker ileal conduit (a urinary diversion created from a segment of the ileum) created a surgical interface between the portal-drained mesenteric circulation and the systemic venous circulation of the abdominal wall.

When portal hypertension arises - in this patient due to chronic liver disease and hepatocellular carcinoma - the pressure gradient forces blood through these small collateral vessels. Over time, these vessels dilate, leading to the formation of high-pressure varices just beneath the thin stomal mucosa. This results in the clinical sign known as the "caput medusae" of the stoma, characterized by a bluish or purple hue. These vessels are extremely fragile, and even minor trauma from a stoma appliance can trigger "voluminous" and life-threatening hemorrhage.

Because of the high mortality rate associated with these bleeds, treatment must be immediate and definitive. Peristomal variceal management can range from conservative therapy to surgical and minimally invasive therapies.

## Introduction

Portal hypertension, most commonly arising from cirrhosis, frequently manifests as variceal bleeding at sites of portosystemic communication. While clinicians are experienced in managing gastroesophageal varices, ectopic varices - those occurring in less common locations - represent a diagnostic and therapeutic challenge. Ectopic varices account for only 5% of all variceal hemorrhages but are associated with a disproportionately high risk of severe morbidity due to their unexpected locations and the difficulty in achieving rapid hemostasis [1].

A particularly rare and precarious subset of these ectopic vessels is peristomal varices, which occur at the junction of a surgical stoma and the abdominal wall. In patients who have undergone a Bricker ileal conduit for bladder malignancy, the creation of the stoma establishes a direct interface between the portal-drained mesenteric veins of the ileal segment and the systemic venous system of the abdominal musculature. Under the pathological pressure gradients of portal hypertension, blood is diverted through these normally dormant collateral pathways. This hemodynamic shift leads to the progressive dilation of thin-walled vessels located immediately beneath the stomal mucosa, a clinical sign colloquially referred to as "caput medusae" of the stoma. Because these vessels are positioned superficially, they are exceptionally vulnerable to friction and minor mechanical trauma, for example, from routine stoma appliance care. Given the high mortality rate associated with stomal hemorrhage, understanding the interventional radiology options - such as percutaneous transhepatic embolization - is critical for managing patients who are poor candidates for more invasive shunting procedures [1,2].

## Case presentation

In this case, we present a male patient with recurrent, voluminous peristomal venous hemorrhage, significant anemia (8.4 g/dL), and visible purple discoloration of the skin surrounding the stoma ("caput medusae" of the stoma) [3]. History includes radical cystoprostatectomy with Bricker ileal conduit for urothelial carcinoma, alongside hepatocellular carcinoma secondary to chronic liver disease.

Contrast-enhanced CT was done, where peristomal varices (dilatation of peristomal vessels) were seen without active extravasation (Figure 1), accompanied by perifocal edema and ascites, due to suspected portal hypertension.

**Figure 1:**
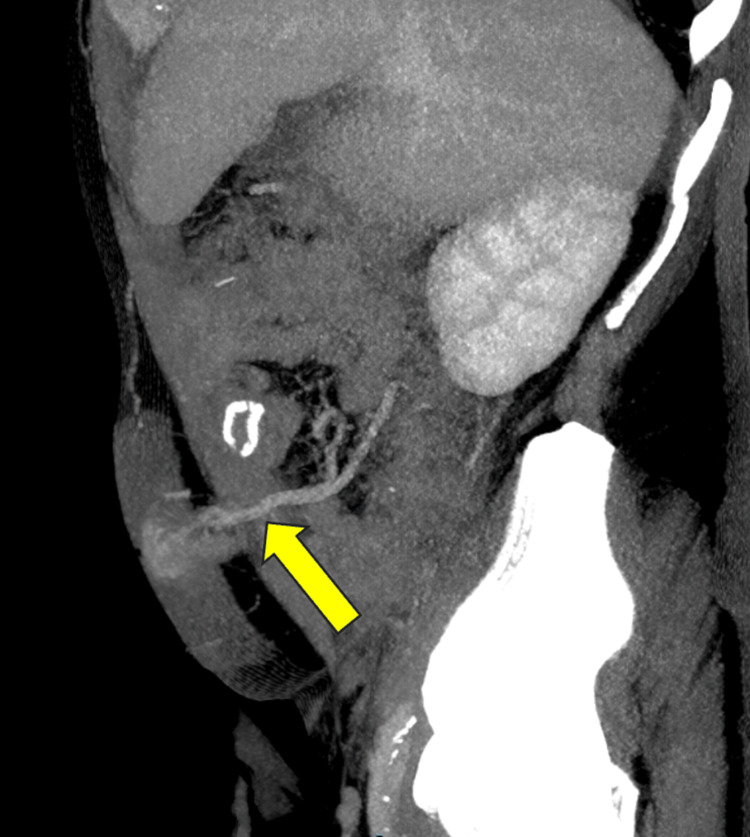
Sagittal contrast-enhanced CT CT image demonstrates a prominent, dilated branch of the mesenteric vein (yellow arrow) providing the primary inflow to a complex of ectopic peristomal varices.

The patient was referred to interventional radiology for peristomal varices embolization to control the bleeding. Access was obtained via a percutaneous transhepatic approach through the right portal vein. Mesenteric venography with a 4 F catheter was performed, and it confirmed the presence of ectopic ileal conduit varices (Figure 2).

**Figure 2:**
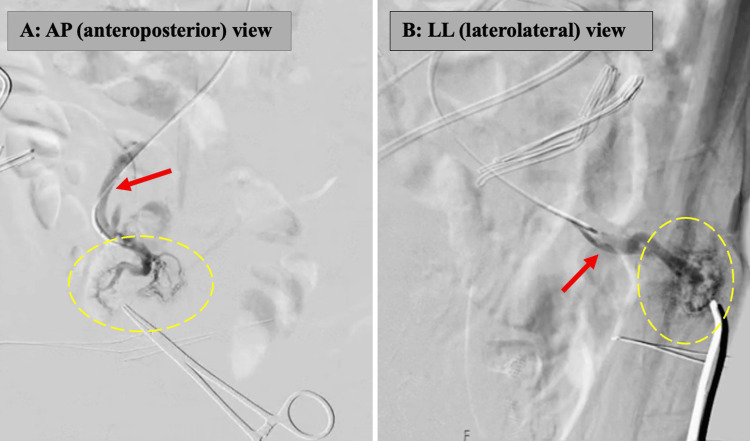
Fluoroscopy venography AP (anteroposterior) and LL (laterolateral) views Anteroposterior (A) and lateral (B) fluoroscopic projections during mesenteric venography reveal a dense network of dilated peristomal varices (yellow circle) arising from a dilated mesenteric vein branch (red arrow). A Kelly clamp is placed externally on the patient's skin to provide a radiopaque landmark, precisely correlating the internal vascular anatomy with the external site of hemorrhage.

The peristomal varices were successfully embolized using coils 7-9 mm in diameter through a 2.7 F microcatheter. Post-embolization venography demonstrated good obliteration of the target variceal branch (Figure 3). At the end of the procedure, the transhepatic puncture tract was embolized with coils to prevent bleeding from the access site. A follow-up Doppler ultrasound performed two days post-procedure confirmed stasis in the dilated peristomal varices, once again indicating a successful technical outcome.

**Figure 3:**
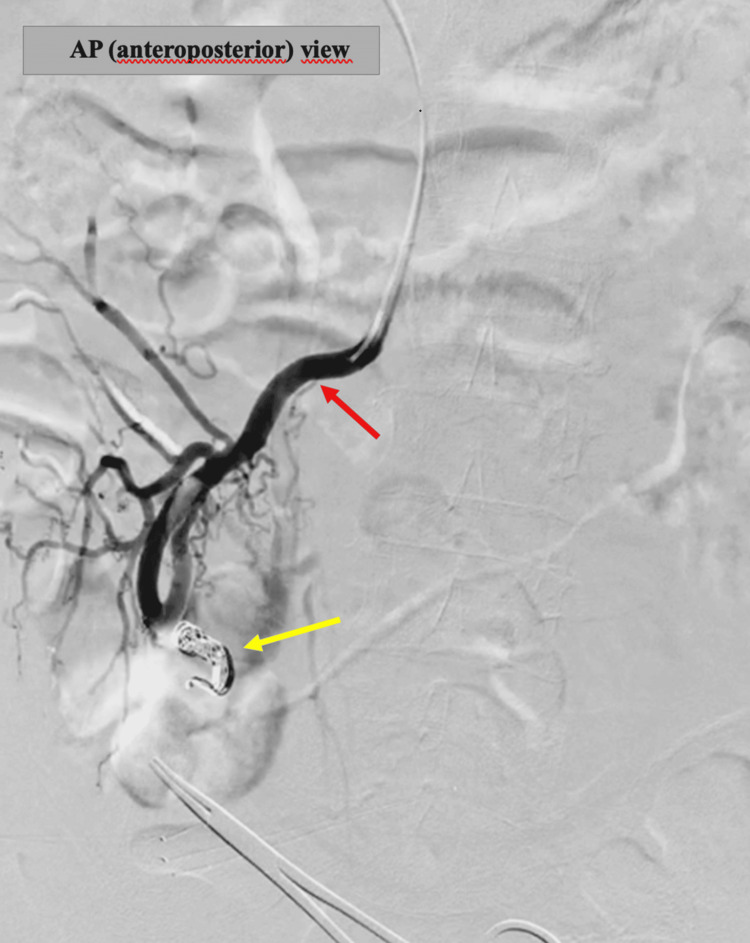
Fluoroscopy AP (anteroposterior) view post-embolization Final fluoroscopic venography demonstrating complete obliteration of the target variceal branches arising from the dilated mesenteric vein branch (red arrow) following coil deployment (yellow arrow). The absence of contrast opacification in the previously dilated peristomal vessels confirms technical success.

The patient’s recovery was uneventful, with instant control of stomal bleeding. He was successfully discharged within days of the intervention. Subsequent monitoring confirmed sustained hemostasis and no recurrent variceal hemorrhage.

## Discussion

Peristomal variceal hemorrhage is a critical clinical event with a mortality rate of 3% to 4% per bleeding episode, demanding immediate and definitive treatment [2]. A standard practice has not yet been established despite the severity of this condition. Current therapeutic strategies range from conservative local therapy to invasive surgical and radiological interventions.

While acute bleeding can be temporarily controlled with wound care and pressure dressings, these localized treatments fail to address the underlying portal hypertension [4]. Surgical reconstruction of the stoma is an option but carries high recurrence rates and perioperative risks. Currently, minimally invasive radiological procedures are preferred for managing ectopic varices. Notably, the creation of a transjugular intrahepatic portosystemic shunt (TIPS) to reduce portal pressure is considered the preferred first-line intervention. However, in cases where TIPS is contraindicated or technically infeasible, percutaneous transhepatic or direct percutaneous vein embolization serves as a highly effective alternative [1,5].

In this case, the transhepatic approach was selected despite the presence of ascites and a hepatocellular carcinoma nodule. To mitigate procedural risks, a paracentesis was performed prior to the transhepatic puncture. By ensuring no free fluid was visible around the liver at the time of the procedure, the risk of intraperitoneal hemorrhage was minimized, making the transhepatic embolization safe to perform.

Post-procedural bleeding after transhepatic portal vein access has been reported in up to 30% of cases when the access tract is left unoccluded, and the risk could be higher in the presence of ascites. However, this risk significantly decreases (up to 6.5%) when the parenchymal tract is closed using Gelfoam or microcoils [2]. As mentioned above, in this case, microcoils were used to close the access site, and no post-procedural bleeding was recorded.

While emergency percutaneous transhepatic coil embolization of bleeding peristomal varices serves as a safe and effective life-saving option - particularly when a TIPS is contraindicated - the long-term threat of recurrence remains a significant limitation of local interventions. As noted in the literature, a prominent limitation of this targeted approach is that additional varices or secondary venous collaterals may still develop downstream due to the persistent underlying portal hypertension [1]. For eligible candidates, TIPS placement should be strongly considered, as it directly targets the root cause of portal hypertension and lowers the rate of stomal variceal rebleeding [6].

## Conclusions

Peristomal variceal hemorrhage in a patient with an ileal conduit is a rare but life-threatening manifestation of portal hypertension. This case highlights the critical importance of maintaining a high clinical suspicion when presented with stomal bleeding in patients with underlying chronic liver disease. Contrast-enhanced CT and venography remain the gold standard for mapping the complex portosystemic collateral anatomy and guiding therapeutic intervention.

While traditional management strategies vary, this case underscores that percutaneous transhepatic coil embolization is a highly effective, minimally invasive alternative to more intensive procedures such as TIPS or high-risk surgical revisions. By achieving definitive hemostasis through targeted occlusion of the mesenteric feeder branches, clinicians can provide a durable solution for patients who may not be suitable candidates for systemic portal pressure reduction. Ultimately, a multidisciplinary approach involving hepatology and interventional radiology is paramount to improving outcomes and reducing the high morbidity associated with ectopic variceal bleeding.
